# Decoding dental mesenchymal stem cells diversity: single-cell transcriptomics maps heterogeneity in molar development

**DOI:** 10.3389/fcell.2025.1712688

**Published:** 2025-12-09

**Authors:** Ruilin Zhang, Leyi Chen, Wanli Xu, Wenan Xu, Buling Wu

**Affiliations:** 1 Nanfang Hospital, Southern Medical University, Guangzhou, Guangdong, China; 2 School of Stomatology, Southern Medical University, Guangzhou, Guangdong, China; 3 Shenzhen Clinical College of Stomatology, School of Stomatology, Southern Medical University, Shenzhen, Guangdong, China; 4 Shenzhen Stomatology Hospital (Pingshan) of Southern Medical University, Shenzhen, Guangdong, China

**Keywords:** dental papillae, dental follicle, single-cell RNA-seq, gene expression, dental mesenchymal cells, differentiation

## Abstract

The spatial and temporal differences influence the development of vertebrate teeth in specific cell types, as well as the precise regulation of signalling networks. During early embryogenesis, the odontogenic potential shifts from the dental epithelium to the mesenchyme, initiating subsequent morphogenetic processes. Across the bud, cap, and bell stages, incisors and molars undergo distinct morphological and functional transformations driven by dynamic mesenchymal subpopulations. These subpopulations exhibit temporally specific gene expression profiles and differentiation trajectories, which orchestrate crown-root patterning, odontoblast differentiation, and pulp-stroma interactions. Recent advances in single-cell RNA sequencing (scRNA-seq) have revolutionised our understanding of dental mesenchymal heterogeneity, unveiling previously unidentified progenitor populations and their regulatory networks. By mapping developmental trajectories and intercellular communication, scRNA-seq has elucidated the transition of mesenchymal cells between stat dental papilla precursors, follicle progenitors, and apical papilla stem cells. Furthermore, this technology highlights the functional divergence of mesenchymal stem cells (MSCs) in postnatal teeth, which balance mineralisation, immune modulation, and repair capacities. However, *in vitro* expansion of MSCs alters their native properties, underscoring the importance of niche-specific signaling. This review synthesises scRNA-seq findings to review the hierarchy of dental mesenchymal subpopulations, offering insights into their roles in developmental defects and regenerative strategies. These discoveries bridge developmental biology and clinical applications, paving the way for novel therapies in tooth regeneration and pulp repair.

## Introduction

Research in embryonic development and stem cell biology is at the forefront of pioneering tissue and organ regeneration. During the early stages of embryonic development, cranial neural crest cells (CNCCs) migrate extensively from the neural tube closure site and differentiate into various cell types, including dental mesenchyme (LaBonne and Bronner-Fraser, 1999; Chen et al., 2015). This mesenchyme subsequently generates two distinct cell populations: the dental papilla, which forms dentin and pulp tissues, and the dental sac, responsible for developing the periodontal ligament, cementum, and alveolar bone (Linde and Goldberg, 1993; Cho and Garant, 2000; Dupin and Sommer, 2012; Rothová et al., 2012). The complex process of tooth development, encompassing the differentiation of the papilla and follicle to the formation of crown and apical structures, provides an illustrative example of the stages involved (Jing et al., 2022). The molecular mechanisms that drive tooth formation are invaluable for understanding embryonic morphogenesis, the onset of function, and the self-renewal capabilities of adult tissues (Yu and Klein, 2020). Moreover, due to their regenerative potential, teeth serve as an exemplary model for organ regeneration, presenting viable paths for future human replacement therapies (Tucker and Sharpe, 2004; Nakao et al., 2007; Ikeda et al., 2009).

Stem cell therapy is at the vanguard of modern approaches to treating various human diseases (Mousaei Ghasroldasht et al., 2022). Dental mesenchymal stem cells (MSCs) can be isolated from various parts of the tooth, including stem cells from human exfoliated deciduous teeth (SHEDs), dental pulp stem cells (DPSCs), periodontal ligament stem cells (PDLSCs), dental follicle stem cells (DFSCs), and stem cells from apical papilla (SCAPs). These cells demonstrate limited differentiation capabilities, generally to only a few cell types, and cannot generate a complete tooth structure on their own, as tooth formation requires reciprocal epithelial–mesenchymal interactions that orchestrate odontogenesis. (Hu et al., 2022; Sonoyama et al., 2008; Chen et al., 2020; Seo et al., 2004; Miura et al., 2003; Gronthos et al., 2000; Morsczeck et al., 2005; Xu et al., 2024). In contrast, dental mesenchyme derived from embryonic tooth germ exhibits more extensive differentiation potential, underscoring the importance of understanding these pivotal stem cell populations within the tooth germ (Hu et al., 2022). This knowledge is crucial for advancing dental tissue regeneration and developing new therapeutic strategies (Nakao et al., 2007; Zhang and Yelick, 2021; Lin et al., 2022; Mina and Kollar, 1987; Yelick and Sharpe, 2019; Cai et al., 2013; Cai et al., 2007).

Over the past 2 decades, advances in transcriptional profiling, particularly with the advent of single-cell RNA sequencing (scRNA-seq), have revolutionised our understanding of cellular dynamics during embryonic development (Ayturk, 2019). Unlike bulk RNA-seq, which averages gene expression across multiple cells, scRNA-seq allows for the detailed examination of gene expression at the individual cell level (Miller et al., 2017; Heyer et al., 2019; Stark et al., 2019). This capability is critical in identifying subtle transcriptional variations and previously unrecognized cell types and states, which might be obscured in bulk analysis (Lähnemann et al., 2020; Tang et al., 2009). ScRNA-seq has revealed the continuous temporal changes in cell types during development, offering a comprehensive view of these dynamics across the entire regulatory network. By employing clustering analysis, scRNA-seq enables the grouping of cells into subpopulations based on distinct gene expression patterns, thus facilitating the identification and characterization of diverse cell states. This approach is augmented by powerful tools such as Cre-engineered mice for selective knockdown and cell tracking and staining methods like fluorescence *in situ* hybridization (FISH) or multiplex immunohistochemistry (mIHC) (Jing et al., 2022).

By integrating public datasets, we aim to deepen our understanding of the intrinsic mechanisms that govern these cells across various developmental stages and locations.

## Odontogenic potential shifts from dental epithelium to mesenchyme

During the early bud stage in mice, the odontogenic potential transitions from the dental epithelium to the dental mesenchyme. This shift enables the mesenchyme to induce non-dental epithelium to participate in tooth development (Mina and Kollar, 1987; Thesleff et al., 1995; Ruan et al., 2018). Notably, studies have successfully formed tooth-like structures by reorganizing the interdental mesenchyme with non-dental epithelium following the early bud phase (Huang et al., 2009; Angelova Volponi et al., 2013). For instance, combining rhesus keratinocyte stem cells with the dental pulp mesenchyme of E13.5 mice, followed by subrenal culture, resulted in the differentiation into enamel-secreting ameloblasts (Ruan et al., 2018). Since the 1970s, when researchers observed that teeth structures formed by recombinant dental mesenchyme from the bud to bell stages of tooth germ development with dental or non-dental epithelium were not significantly different from those developed normally, there has been ongoing exploration into which key molecules regulate the odontogenic potential of mesenchyme after it is transferred from the epithelium (Kollar and Baird, 1970a; Kollar and Baird, 1970b). Given this unique biological phenomenon of odontogenic potential conversion during the early bud stage, Yaofeng Wang et al. (Wang et al., 2022) explored the gene expression changes before and after this transition. These genes are silent in dental mesenchymal cells before the bud stage but are actively expressed afterwards, including *Enpp1, Gldn, Hivep2, Htra3, lgfbp3, Lgals7, Loxl1, Lypd1, Plac8, Dlx4, Dlx4os, Kazald1, Adamts2,* and *Thy1*. *ENPP1* is important in mineralisation and bone tissue remodeling (Mackenzie et al., 2012; Hajjawi et al., 2014; Roberts et al., 2019). Mutations in *ENPP1* lead to hypercementosis, highlighting its role in maintaining proper mineral balance and tissue homeostasis (Thumbigere-Math et al., 2018; Ferreira et al., 2024). Thegene represents a functional shift not only for dentinogenesis but also for potential therapeutic strategies targeting mineralization defects in dental tissues. *Lypd1* is a glycosylphosphatidylinositol-anchored protein and can regulate odontoblast differentiation during tooth development (Fu et al., 2023). The knockout of *Lypd1* inhibits odontoblast differentiation, emphasizing its pivotal role in this transitional phase. Changes in *Lypd1* expression pattern provide insights into modulating odontoblast differentiation for regenerative dental therapies. Multiple members of the *Dlx* gene family, such as *Dlx1, Dlx2, Dlx3, and Dlx4*, have been reported to participate in mammalian tooth development (Ramanathan et al., 2018; Hii et al., 2023). The *Dlx* genes serve as a functional switch that directs mesenchymal cells towards a specialized osteoblast lineage, making them a valuable target for tissue engineering strategies (Zhang et al., 2019; Ye et al., 2012). The regeneration of human tissues or organs has long been a goal we strive to achieve. Researchers have identified that *Kazald1* in salamanders may be a key gene regulating limb regeneration (Bryant et al., 2017). Although its role in tooth development remains to be fully elucidated, Kazald1 represents an interesting candidate for further investigation. *Adamts2* plays a role in cranial-facial development, and inhibiting *Adamts2* can disrupt the FrbB pathway, subsequently suppressing osteogenesis (Yao et al., 2022).

## The fate diversity of dental mesenchymal cells

CNCCs migrate into the oral region of the first pharyngeal arch, giving rise to a multipotent dental mesenchymal lineage. This lineage subsequently differentiates into the dental follicle and papilla lineages. The dental follicle differentiates into periodontal tissues, while the dental papilla gives rise to odontoblasts and dental pulp (Krivanek et al., 2017; Cobourne and Sharpe, 2003).

### Heterogeneity of mesenchymal cells in molars and incisors

The growth patterns of incisors and molars in mice are different (Table 1): while mouse incisors continue to grow throughout their lives, mouse M, similar to human molars, do not exhibit lifelong growth. This continuous growth in mouse incisors is facilitated by perpetually dividing and self-renewing epithelial and mesenchymal stem cells, compensating for tissue loss due to repetitive gnawing. Consequently, mouse incisors serve as a model for studying organs with sustained self-renewal, providing insights into the mechanisms regulating growth, development, and adult stem cell biology (Pugach and Gibson, 2014; Seidel et al., 2017; Lacruz et al., 2017; Feng et al., 2011; Harada et al., 1999; Harada and Ohshima, 2004). Due to the similarity between mouse and human molars and the importance of mouse M in studying organ self-renewal, both mouse M and incisors have become widely used models for human studies.

**TABLE 1:** Heterogeneity of mesenchymal cells in molars and incisors.

Category	Incisor (mouse)	Molar (mouse)	Functional/interpretive notes	Refs
Growth dynamics	Continuous growth throughout life via self-renewing epithelial/mesenchymal stem cells	No lifelong growth; resembles human molars	Incisors as a model of sustained self-renewal; molars model limited growth typical of humans	Pugach and Gibson (2014), Seidel et al. (2017), Lacruz et al. (2017), Feng et al. (2011), Harada et al. (1999), Harada and Ohshima (2004)
Time-window gene programs (E12.5–E16.5)	*Pax3* (E12.5–E14.5); *Tfap2a, Sfrp4, Alx1/Alx3* (E12.5–E16.5); *Hand1* (E12.5)	*Tfap2b, Lhx6* (E12.5–E16.5); *Tbx15, Sfrp2* (E12.5–E14.5)	Distinct temporal programs imply lineage/pathway divergence between tooth types; mechanisms underlying timing remain unclear	Wang et al. (2022), Monsoro-Burq (2015)
Stem-cell niche	Persistent niche at apical root; regenerative/hard-matrix genes in apical pulp: *Lef1, Sgrp2, Fzzd1*, *Rspo1, Sfrp1, Gli1, Trabd2b, Wif1*	These niche-associated gene signatures are rare	Apical incisor niche supports ongoing renewal and injury-responsive matrix production	Hu et al. (2017), Krivanek et al. (2020)
Mesenchymal compartment composition (adult)	Odontoblasts; apical pulp subtypes (*Smoc2* ^*+*^ *, Sfrp2* ^*+*^) incl. Quiescent stem pools; supportive stroma; incrementally differentiating distal pulp (*Igfbp5* ^*+*^ *, Syt6* ^*+*^)	Distal incisor mesenchyme resembles molar mesenchyme—both largely terminally differentiated	Regional specialization across incisor; distal domain convergence with molar mesenchyme	Krivanek et al. (2020)
*PRX1* ^*+*^ lineage contribution	—	*PRX1* ^*+*^ cells contribute to M1 development; differentiate into odontoblast progenitors, fibroblasts	Stage-dependent *PRX1* ^*+*^ contribution; aligns with timing differences among molars	Gong et al. (2022), Higuchi et al. (2015)
*PRX1* ^*+*^ cells in periodontal ligament (PDL)	—	*PRX1* ^*+*^ cells abundant in PDLCs; co-localize with perivascular cells; promote angiogenesis during PDL development and after injury	Perivascular *PRX1* ^*+*^ cells support vasculature and repair, linking mesenchyme to vascular niche	Gong et al. (2022), Higuchi et al. (2015), Bassir et al. (2019), Douville and Wigle (2007), Frenette et al. (2013)
Trajectory differences (pseudotime)	—	Epithelial lineage ∼ linear from E12.5–PN7; mesenchyme exhibits branched fates; epithelial differentiation low at E12.5–E16.5, rises postnatally; mesenchymal differentiation high postnatally (E16.5 > E12.5–E14.5)	Epithelial vs. mesenchymal dynamics diverge; mesenchyme shows early branching potential	Hu et al. (2022)
Knowledge gap	Mechanistic basis for incisor-specific timing remains unresolved	Mechanistic basis for molar-specific timing remains unresolved	Reasons/mechanisms behind time-window and tooth-type specificity are under-investigated	—

During E12.5 to E16.5, dental mesenchymal cells of mice molars and incisors exhibit distinct pathways and marker genes. Members of the *PAX* gene family, as highly conserved transcription regulators, have been shown to play a crucial role in the development of neural crest (Monsoro-Burq, 2015). For the incisors, *Pax3* is specifically expressed from E12.5 to E14.5, *Tfap2a, Sfrp4, Alx1,* and *Alx3* are specifically expressed at E12.5 to E16.5, and *Hand1* is specifically expressed at E12.5. In molars, *Tfap2b* and *Lhx6* are expressed at E12.5 to E16.5, whereas *Tbx15* and *Sfrp2* are expressed at E12.5 to E14.5 (Wang et al., 2022). However, the reasons and mechanisms behind the expression of these genes during specific time windows and in particular tooth types have not been thoroughly investigated.

**FIGURE 1:**
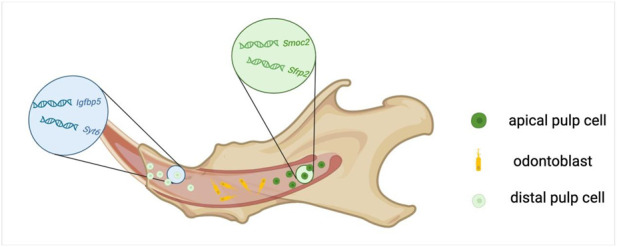
Cellular composition of the dental pulp in mouse incisors.

A continuously renewing stem cell niche is presented at the root of the incisor in mice (Hu et al., 2017). Genes such as *Lef1, Sgrp2, Fzzd1, Rspo1, Sfrp1, Gli1, Trabd2b,* and *Wif1*, which are associated with the regenerative response and the production of hard matrix after physical injury, are expressed in the pulp of the apical incisor and are rarely found in the molars (Krivanek et al., 2020). Krivanek et al. suggest that the mesenchymal compartment in the pulp chamber of adult mouse incisors comprises odontoblasts, apical pulp subtypes labeled with *Smoc2* and *Sfrp2*, including various quiescent stem cell pools, and supportive stromal cells, and incrementally differentiating distal pulp cells, labeled with *Igfbp5* and *Syt6* (Figure 1) (Krivanek et al., 2020).

The distal mesenchyme of mice incisors and the mesenchyme of molars exhibit similarities, both consisting of terminally differentiated populations (Krivanek et al., 2020). *Prx1+* mesenchymal cells and their progeny are involved in the development of the first molar (M1), and Prx1+ cells can differentiate into a variety of cell types, including odontoblast progenitor cells, fibroblasts, and dental pulp cells during cytodifferentiation stages (Gong et al., 2022; Higuchi et al., 2015). Likely due to the different developmental time points, there are differences in the distribution proportion of *PRX1+* cells in M1, M2, and M3, with their distribution in the dental pulp decreasing sequentially. In addition, *Prx1+* cells are abundantly distributed in PDLCs, co-localizing with perivascular cells and contributing to angiogenesis during PDL development and after injury repair (Gong et al., 2022; Bassir et al., 2019; Douville and Wigle, 2007; Frenette et al., 2013).

Pseudotime analysis indicates that there were differences in the differentiation trajectories of epithelial and mesenchymal cells in mouse M (Hu et al., 2022). From E12.5 to postnatal day 7 (PN7), the differentiation trajectory of epithelial cells is linear, whereas mesenchymal cells undergo various differentiation fates, presenting a branched differentiation pattern. The differentiation degree of epithelial cells is relatively low, from E12.5 to E16.5, but increases significantly after birth. Although mesenchymal cells also exhibit a high degree of differentiation after birth, E16.5 was more differentiated than E12.5 to E14.5.

### Cell heterogeneity during molar papilla and follicle development

#### Bud stage

**FIGURE 2:**
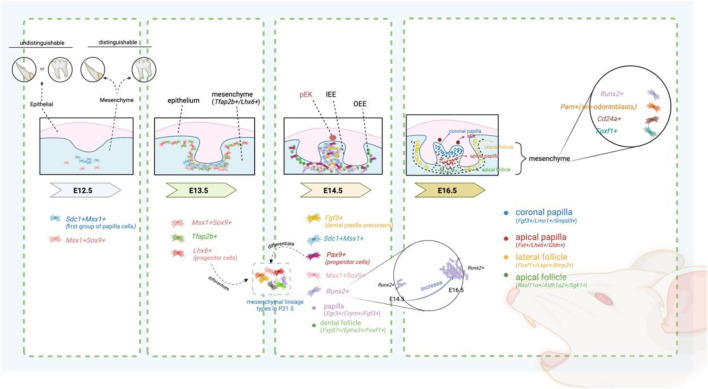
Spatio-temporal changes in specific subtypes during the development of mesenchymal tissue in mouse M pEK: primary enamel knots; sEK: secondary enamel knots.

Wang et al. have demonstrated that molars and incisors in mice can be distinguished only after E12.5, and this distinction is observed exclusively in the dental mesenchyme through single-cell sequencing (Figure 2) (Table 2) (Wang et al., 2022). Significant differences in markers, regulons, and signaling pathways in the mesenchyme of molar and incisor suggest that this period is considered a transfer stage of odontogenic potential. The development of mesenchymal cells in incisors and molars is stagnant from E13.5 to E14.5 through pseudotime analysis. At E13.5, dental mesenchymal cells, which can be labelled with Tfap2b+/Lhx6+, are relatively homogeneous, with the *Lhx6+* cell population representing the dental mesenchymal progenitor cells capable of forming all dental mesenchymal lineage types by P21.5 (Figure 2) (Jing et al., 2022). Interestingly, despite being specifically expressed in the mesenchyme both before and after mouse birth, the expression level of *Lhx6* gradually decreases from PN0.5 to PN7.5 postnatally (Grigoriou et al., 1998; Denaxa et al., 2009). From the perspective of cellular subpopulations, *Lhx6+* cells are found within *Gli1+* cells. *Gli1+* mesenchymal cells are a heterogeneous population of progenitor cells associated with root development in teeth (He et al., 2021). Specifically, *Lhx6+* cells represent a subset of *Gli1+* root dental progenitor cells (He et al., 2021). From the standpoint of cellular localization, from E13.5 to E14.5, the expression pattern of *Lhx6* in dental mesenchyme is uniform and does not exhibit specific spatial localization. However, starting from E16.5, when the developmental baton is about to be passed from the crown to the root, *Lhx6* begins to exhibit spatial distribution characteristics, primarily expressed in the mesenchymal cells of the apical region. Functionally, *Lhx6+* cells have the potential to differentiate into *Dspp*-marked odontoblasts and are closely associated with root development (He et al., 2021; Jing et al., 2019). The molecular mechanism of odontoblast differentiation from progenitor cells involves *Lhx6* inhibiting *Sfrp2*, which in turn inhibits *Wnt10a*, thereby promoting the activation of the Wnt signaling pathway and facilitating the differentiation of progenitor cells into odontoblasts (He et al., 2021). The formation of the first group of papilla cells, marked with *Sdc1+Msx1+*, first appearing beneath the epithelium, also begins at E12.5 (Hu et al., 2022). These dental papilla cells labelled with *Sdc1+Msx1+* also proliferate and expand between E13.5 and E14.5, forming the dental papilla. However, these cells are no longer present in the P1 mesenchyme (Hu et al., 2022).

**TABLE 2:** Cell heterogeneity during molar papilla and follicle development.

Stage	Time	Cell type	Location	Characteristic	References
Bud	∼E12.5	—	—	Molars and incisors can be distinguished by the difference of mesenchymal	Wang et al. (2022)
	E12.5	*Sdc1+*mesenchymal cells	Dental papilla	The first group of papilla cells	Hu et al. (2022)
	E12.5	*Msx1+Sox9+* niche cells	Dental niche	A group of stem cells with the ability to reconstruct teeth and directly contribute to dental papilla	Hu et al. (2022), Aberg et al. (2004b)
	E13.5	*Lhx6+* mesenchymal cells	Dental mesenchymal	Progenitor cells, which are capable of forming all dental Mesenchymal lineage types by P21.5	Jing et al. (2022)
	E13.5∼E14.5	—	—	arrest of mesenchymal development	Wang et al. (2022)
Cap	E14.5	*Fgf3+* mesenchymal cell	Dental papilla region	Dental papilla precursors	Hu et al. (2022), Kettunen et al. (2000)
	E14.5	*Crym+/Egr3+/Fgf3+*	Dental papilla	Regional marker	Jing et al. (2022)
	E14.5	*Epha3+/Fxyd7+/Foxf1+*	Dental follicle	Regional marker	Jing et al. (2022)
Bell	E16.5	*Fgf3+/Lmo1+/Smpd3+*	Crown papilla	Regional marker	Jing et al. (2022)
	E16.5	*Fst+/Lhx6+/Gldn+*	Apical papilla	Apical papilla	Jing et al. (2022)
	E16.5	*Foxf1+/Lepr+/Bmp3+*	Lateral follicle	This dental sac pattern remains unchanged until P3.5	Jing et al. (2022)
	E16.5	*Rasl11α+/Aldh1α2+/Sgk1+*	Apical follicle	This dental sac pattern remains unchanged until P3.5	Jing et al. (2022)
	E12.5∼E16.5	*Runx2+* mesenchymal cells	Dental mesenchyme	First appeared in the condensed mesenchyme in the early bud stage,then in the tooth papilla until the early bell stage, and then in the dental follicle	Hu et al. (2022), Aberg et al. (2004b), Zhang et al. (1997), D'Souza et al. (1999)
	E16.5	*Pam +* mesenchymal cells	Dental mesenchyme	Possibly represent pre-odontoblasts	Hu et al. (2022), Aberg et al. (2004b), Zhang et al. (1997), D'Souza et al. (1999)
	E16.5	*Lepr +* mesenchymal cells	Lateral sac	The progenitor cells of the dental follicle	Jing et al. (2022)
	E16.5	*Barx1+* mesenchymal cells	Dental papilla	Strongly expressed	Wang et al. (2022)
	E16.5	*Barx1+* mesenchymal cells	Dental follicle	Weakly expressed	Wang et al. (2022)
	E16.5	*Cd24+*mesenchymal cells	Dental papilla	*Cd24++* cells are spatially distributed in the upper part of the dental papilla, forming an irregularly thick cap, and *Cd24+* cells are located in the lower part of the papilla	Wang et al. (2022)
	E16.5	*Plac +* mesenchymal cells	Spatially distributed at the top layer of dental papilla and slightly in the dental follicle	Having the potential to induce tooth regeneration	Wang et al. (2022)
After birth	P1	*Creb3l1+* mesenchymal cells	Dental mesenchyme	First appearance	Hu et al. (2022)
	P1	*C1qtnf3+* and *Epha7+* mesenchymal cells	Dental mesenchyme	First disappearance	Hu et al. (2022)
	P1	*Ccna2+* mesenchymal cells	Dental mesenchyme	a) Noticeable reduction b) Associated with the loss of interstitial rebuilding capacity in teeth	Hu et al. (2022)
	P3.5	*Fabp7+/Enpp6+* cells	Coronal papilla	a) Derived from the differentiation of dental papilla cells b) The apical papilla cells are found to be the bipotent progenitor cells that can differentiate into odontoblast and pulp lineages	Jing et al. (2022)
	P3.5	*Rab3b+/Nnat +* cells	Middle papilla		Jing et al. (2022)
	P3.5	*Tac1+/Aox3+* cells	Apical papilla		Jing et al. (2022)
	P3.5	*lfitm5+/Phex +* cells	Odontoblasts		Jing et al. (2022)
	P3.5	*Tnmd+/Bmp3+* cells	Lateral dental follicle	a) Derived from the differentiation of dental sac cells b) The apical dental follicle controls the lineage development of root furcation region	Jing et al. (2022)
	P3.5	*Slc1a3+/Smoc2+* cells	Apical dental follicle		Jing et al. (2022)
	P7	*Enpp6+*mesenchymal cells	Dental mesenchyme	First appearance	Hu et al. (2022)

During this period, researchers have found that mesenchymal cells not only secrete autocrine signals to themselves but also provide key signals to other mesenchymal cells and epithelial cells, such as periostin, MK, CXCL, and PTN, as revealed through CellChat analysis (Hu et al., 2022). There are also numerous outgoing and incoming signals between the epithelial and mesenchymal cells, indicating a complex network of intercellular communication during this critical developmental stage.

#### Cap stage

At E14.5, the first important sign of the diversification of dental mesenchymal cell lineages in the process during mouse tooth development is the separation of dental follicle and dental papilla, both derived from the mesenchymal cells (Jing et al., 2022). Hong Hu et al. further identified *Fgf3+* mesenchymal cells at E14.5, which are likely dental papilla precursors (Figure 2) (Hu et al., 2022; Kettunen et al., 2000). *Fgf3* expression in the mesenchyme during tooth development is dependent on the expression of *Msx1* and *Runx2* (Aberg et al., 2004a). *Fgf3* and *Fgf10* play a crucial role in regulating molar size through a synergistic action (Kwon et al., 2015; Wang et al., 2007). This synergy is, to some extent, redundant (Wang et al., 2007). The understanding of the origin of the papilla has evolved, initially positing that most of the mesenchymal cells formed the papilla, while only a small group of cells surrounding the enamel organ and papilla form the dental follicle (Tucker and Sharpe, 2004; Chai et al., 2000; Aberg et al., 2004b; Takatalo et al., 2009). It was later found that the papilla is formed only by the mesenchymal cells adjacent to the inner dental epithelium and between the enamel organ, with the remaining mesenchymal cells forming the dental sac (Rothová et al., 2012).

Interestingly, there is also a flow of cells between the tooth sac and the papilla, with some dental sac cells moving out during the cap stage and entering the tooth papilla in the bell stage. The development of the papilla may require additional cells from the dental niche. Single-cell sequencing further supplemented and refined this concept, emphasizing the importance of dental niche cells in the process of epithelial-mesenchymal interactions (Hu et al., 2022). Dental niche cells can induce tooth formation by promoting the reorganization and survival of dental epithelial cells, as well as the regeneration of *Msx1+Sdc1+* dental papilla cells (Hu et al., 2022). In both molar E12.5 and E14.5, dental niche cells were found to be in an earlier progenitor state than papilla precursors (Hu et al., 2022). *Msx1+Sox9+* niche cells are a group of stem cells with the ability to reconstruct teeth and directly contribute to dental papilla. These cells appear in the dental niche for the first time at E12.5. Subsequently, these cells gradually increase in number, positioning themselves around the dental papilla by E14.5. After that, they migrate into the papilla at E15.5 to E16.5, completely replacing the papilla cells by E18.5 and P1. Once in the nipple region, migrating dental niche cells activate a morphogenetic network *(RUNX1/STAT3, SP7, RUNX2, CREB3L1*) that reprograms them from motile niche cells into regionally specified mesenchyme with enhanced proliferation/survival, matrix synthesis and secretion, and paracrine signaling capacity, thereby supporting papilla expansion, follicle maturation, vascular/perivascular support, and early root/periodontal patterning (Hu et al., 2022; Aberg et al., 2004b; Andersson et al., 2020; Sarper et al., 2018; Wen et al., 2020; Thumbigere-Math et al., 2019; Bae et al., 2018).

*Pax9+* cells are progenitor cells that can generate all mesenchymal lineages at P21.5 and are distributed in mesenchymal cells at E14.5, including the papilla and dental follicle (Jing et al., 2022). At the cap stage, the dental papilla and the dental follicle surrounding the dental papilla and dental epithelium can be marked with *Egr3/Crym/Fgf3* and *Fxyd7/Epha3/Foxf1*, respectively (Jing et al., 2022). During the earliest stages of tooth development, the expression of *Pax9* in the mesenchyme is dependent on Fgf signaling (Zhou et al., 2011). During tooth development, *Pax9* is highly expressed in the mesenchyme and is necessary for the expression of *Lef1, Msx1,* and *Bmp4* in the mesenchyme (Intarak et al., 2023). Additionally, the expression of *PAX9* can be used to locate the position of tooth development before the morphological manifestation of tooth generation (Peters et al., 1998a). Importantly, the absence of *PAX9* function can lead to developmental abnormalities such as tooth loss and skeletal abnormalities (Peters et al., 1998b). Knockout of *Pax9* in mice results in the arrest of tooth germ development at the bud stage (Peters et al., 1998b). This highlights the critical role of *Pax9* in early tooth development.

In addition, at E14.5—when papilla and follicle lineages separate—SCENIC analysis identifies domain-specific regulons that operationalize this split: papilla regulons (*FOSB, ZMIZ1, FOXN3, CREB1/3/5, RBBP5, PBX1, FOXO3, NR2C2*) coordinate a transition from broadly multipotent mesenchyme to an odontogenic-competent, pre-secretory state by coupling controlled proliferation, epigenetic priming, and polarized secretory/ECM programs; follicle regulons (*EGR3, NRF1, ELK3, ELF1/2/4, FOXO1, SIN3A, SIX5, NR3C1*) reprogram cells toward a fibro-ligament/cementogenic trajectory characterized by mechano- and angiogenic signaling, mitochondrial/metabolic support, chromatin re-organization, and steroid-responsive ECM remodeling. The co-localization of these transcription factors with their target genes within each domain supports cell-autonomous execution of these distinct morphogenetic transitions (Jing et al., 2022).

At 9–10 gestational weeks (gw) in humans, the apical dental papilla (ADP) cluster, defined by *NIFA+/COL14A1+* and *RBMS3+* markers, constituted the predominant cell population within the dental papilla (DP), in contrast to the coronal dental papilla (CDP) population (*FGF3+/TFAP2A+/CPVL+*) (Zhang et al., 2025). This cellular composition underwent a notable transition by 12 PCW, where the proportion of CDP clusters exhibited progressive expansion while ADP representation declined (Zhang et al., 2025). During human tooth germ development at 13 gw, the proportion of dental mesenchymal progenitor cells reaches its peak (Alghade et al., 2023). At this stage, the dental pulp is predominantly composed of DP cells, while the dental ectomesenchyme (DEM) is primarily localized to the apical region of the pulp (Alghade et al., 2023).

#### Bell stage

During the bell stage, significant differentiation occurs within the dental papilla and follicle. The dental papilla differentiates into spatially distinct crown (*Fgf3+/Lmo1+/Smpd3+*) and apical domains (*Fst+/Lhx6+/Gldn+*) (Figure 2). Concurrently, the tooth follicle differentiates into lateral (*Foxf1+/Lepr+/Bmp3+*) and apical domains (*Rasl11α+/Aldh1α2+/Sgk1+*). This dental sac pattern remains unchanged until P3.5 (Jing et al., 2022). Notably, the cluster of mesenchymal *Runx2+* cells significantly increased, and mesenchymal *Pam +* cell clusters appeared, the latter of which is thought to possibly represent pre-odontoblasts (Hu et al., 2022; Zhang et al., 1997). The *Runx2+* cell population is predominantly located at the bottom of the dental follicle at E14.5, with a smaller portion found in the dental papilla (Figure 2) (Hu et al., 2022). *Runx2*, a transcription factor expressed by the mesenchyme, regulates the epithelial-mesenchymal interaction during the bud and cap stages of tooth development (Aberg et al., 2004b; D'Souza et al., 1999). *Runx2* maintains a close regulatory relationship with *Msx1* and *Osr2*. Researchers observed that upon conditional knockout of *Msx1* and *Osr2* in mice, *Msx1* positively and *Osr2* negatively regulate *Runx2* during the early stages of tooth development (Kwon et al., 2015). Additionally, *Runx2* plays a crucial role in the differentiation of osteoblasts and their subsequent osteogenic functions (Zeng et al., 2022; Ducy et al., 1997; Liu and Lee, 2013). Mice with *Runx2* mutations exhibit tooth agenesis and developmental arrest at the bud stage (D'Souza et al., 1999). By individually knocking out *Runx2* and *Osr2* in mice, researchers found that *Osr2* could partially rescue the abnormal molar morphology in *Runx2* knockout mice (Kwon et al., 2015).

**FIGURE 3:**
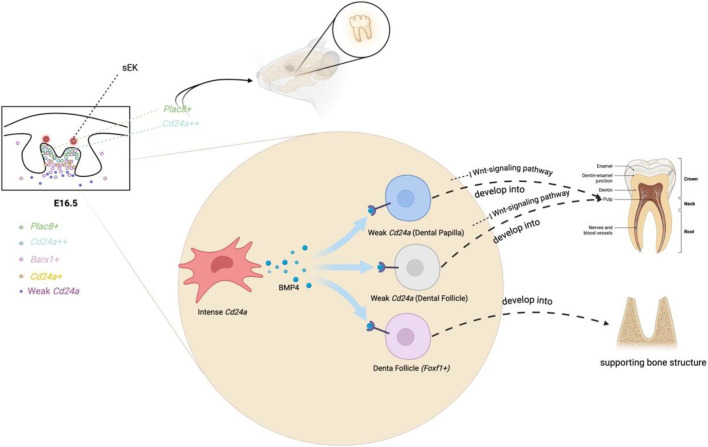
Biological events in the dental mesenchyme at E16.

Throughout the development of incisors and molars, certain genes maintain stable and high expression from E10.5 to E16.5, including *Msx1, Pax9, Bmp4, Lhx8, Foxf1,* and *Cd24a*. These key genes involved in tooth development are mostly closely interconnected. For example, the knockout of *Msx1* results in the arrest of tooth development at the bud stage in mice and also inhibits the expression of *Bmp4* (Chen et al., 1996). By adding *Bmp4*, a crucial signaling molecule during the bud’s advancement to the cap stage, it enables the tooth embryos of *Msx1* knockout mice, which were originally arrested at the bud stage, to successfully pass through the bud and continue their development to the late bell stage (Rothová et al., 2012). *Cd24a++* cells have shown the potential to induce tooth regeneration (Figure 3) (Wang et al., 2022). At E16.5 of the first molars, *Cd24a*-positive cells are all located in the *Barx1*-positive expression area. Barx1 is strongly expressed in the entire dental papilla region but is weakly expressed in the dental follicle region (Figure 3). More precisely, *Cd24++* cells are spatially distributed in the upper part of the dental papilla, forming an irregularly thick cap, while *Cd24+* cells are located in the lower part of the papilla. In addition, the *Barx1* gene regulatory network is highly active in the cap stage. Both *Cd24a* and *Barx1* were highly expressed from E10.5 to E12.5, inhibited from E13.5 to E14.5, and then hyperexpressed again at E16.5. The odontogenic fate of *Cd24a++* cells begins from the bell-shaped stage, marking *Cd24a* as an important odontogenic marker gene in the bell stage of molars (Wang et al., 2022).

On the other hand, Plac8+ cells are also found to have the potential to induce tooth regeneration (Figure 3). At E16.5 of the first molar, *Plac8+* cells, which are highly specific in regulating cell cycling and epithelial cell proliferation, are spatially distributed at the top layer of dental papilla and slightly in the dental follicle (Wang et al., 2022). *Plac8+* and *Cd24a++* exhibit overlapping spatial positions at the apical region of the tooth papilla. *Cd24a++* cells in the upper part of the papilla secrete *Bmp4* to stimulate the weak *Cd24a* region of the lower papilla and the dental follicle. Subsequently, the weak *Cd24a* cells inhibit the Wnt-signaling pathway and differentiate into the pulp, and the dental follicle cells (Foxf1+) also form supporting bone structures in response to BMP signaling (Figure 3) (Wang et al., 2022).

Spatiotemporal transcriptomic profiling reveals distinct architectural patterns during human odontogenesis (Shi et al., 2024). At 17 gw, the primary tooth germ exhibits a polarized cellular organization along the distal-proximal axis: dental follicle cells progressively transition into distal pulp (*SOX9+*), followed by apical pulp (*SFRP1+/SOSTDC1+/SMOC2+*) before terminating at the epithelial interface (Shi et al., 2024). Within the primary tooth germ at 24 gw, spatial mapping revealed *FBN2+* odontoblasts occupying the peripheral pulp zone. Notably, permanent tooth germs display a diffuse distribution of *TNC +* distal pulp cells lacking regional specificity (Shi et al., 2024). At 19 gw during human tooth germ development, the dental papilla (DP) is surrounded by the dental follicle (DF) (Alghade et al., 2023). At this stage, the cellular composition and spatial organization within the DP reveal odontoblasts (OB) localized adjacent to the dental epithelium, with subodontoblasts (SOB) and preodontoblasts (POB) residing beneath in a mixed distribution (Alghade et al., 2023). Given the minor proportion of SOB within the dental pulp, it is hypothesized that POB serves as the primary source of functional OB, while SOB functions as a reserve progenitor pool for dentinogenesis (Alghade et al., 2023).

In summary, the bell stage is characterized by significant differentiation and spatial organization within the dental papilla and follicle, which further enhances the separation of these two parts following the cap stage.

#### After birth

After birth, mesenchymal stem cells gradually differentiate into their terminal phenotypes. Clusters of *Enpp6+* and *Creb3l1+* mesenchymal cells appear, while the vast majority of mesenchymal *C1qtnf3+* and *Epha7+* cell clusters, which are related to tooth reconstruction, disappear. Additionally, there is a noticeable reduction in mesenchymal *Ccna2+* cell clusters (Hu et al., 2022). At P3.5, when root development begins, the papilla cells differentiate into coronal (*Fabp7+/Enpp6+*), middle (*Rab3b+/Nnat+*), apical papilla (*Tac1+/Aox3+*), and odontoblasts (*lfitm5+/Phex+*), which are spatially separated and exhibit different states of differentiation. Concurrently, dental sac cells differentiate into spatially separated lateral (*Tnmd+/Bmp3+*) and apical (*Slc1a3+/Smoc2+*) dental follicles, with the latter controlling the lineage development of the root furcation region. Among them, apical papilla cells are identified as bipotent progenitor cells capable of differentiating into odontoblast and pulp lineages (Jing et al., 2022). Unlike the developmental mechanism, it is the pulp cells of the crown region that form odontoblasts during post-injury restoration (Neves and Sharpe, 2018; Zaugg et al., 2020). *Pthrp +* cells exhibit dynamic spatial redistribution during tooth development (Takahashi et al., 2019). At E15.5 through P3, this cellular population predominantly localizes within the dental follicle surrounding the tooth germ. During post-eruption stages (P25/P18.2), their distribution undergoes substantial reorganization, relocating to the root surface region. Single-cell analysis reveals pronounced heterogeneity within the *Pthrp +* cell cluster at P3, encompassing odontoblast/dental papilla mesenchymal cells, fibroblast subpopulations, epithelial cells, and dental follicle-derived progenitor cells. These findings suggest that the dental follicle microenvironment harbours multilineage regulatory potential during early odontogenesis (Takahashi et al., 2019).

Using CellChat analysis, Jing et al. found that, at P3.5, there are complex interactions between the mesenchymal cells (Jing et al., 2022). In the outgoing signaling pattern, the coronal dental papilla secretes *ANGPTL*, *ncWNT*, *PDGF*, the apical dental papilla secretes *MK, PTN, WNT*, the middle papilla secretes *MK, ncWNT, BMP*, the lateral follicle secretes *ANGPTL, ncWNT, IGF, PTH*, apical follicle secretes *MK, PTN, BMP, PTH* and odontoblast secretes *BMP* and *WNT*. These signals are very important for the proper development of teeth. Taking the example of *Wnt10a*, a very canonical *WNT* ligand secreted by odontoblasts and apical papillae, is essential; its absence in the mesenchyme can lead to defective root development (Tamura and Nemoto, 2016; Zhang H. et al., 2023; Murashima-Suginami et al., 2021; Hermans et al., 2021). In addition, the cell-cell interactions mediated by *Igf1-Igf1r* between the lateral and apical follicle compartments are crucial for the development of PDL (Jing et al., 2022). The postnatal development of dental mesenchymal cells displays increased heterogeneity due to their spatial distribution, constraining the complexity of the tooth structure.

Comparative transcriptomic profiling of human embryonic and postnatal dental mesenchymal cells (marked by *EMILIN1* and *MSX1*) revealed high transcriptional similarity among the dental follicle, odontoblasts, and distal pulp (*SOX9+*), with the exception of the apical pulp compartment (*SFRP1+/SOSTDC1+/SMOC2+*) (Shi et al., 2024).

## Dental mesenchymal cells developing towards terminal differentiation

As teeth erupt and establish occlusion, dental pulp tissue gradually matures, and mesenchymal stem cells (MSCs) increasingly differentiate into terminal cell types. In this section, we focus solely on the mesenchymal components within the dental pulp, which are generally classified into odontoblasts, fibroblasts, and undifferentiated mesenchymal stem cells retained within the pulp tissue.

### Odontoblasts: cells forming dentine

Odontoblasts, though comprising a small fraction of dental pulp cells, play a crucial role in dentinogenesis and immune responses. Originating from cranial neural crest cells, odontoblasts undergo a complex differentiation process regulated by various molecular signals. As described by Wang et al. (Gong et al., 2022), odontoblast differentiation begins early, before these cells are integrated into the odontoblastic layer. Mesenchymal cells near the cervical loop express early markers such as *Notum* and *Sall1*, which initiate the commitment of multipotent mesenchymal cells toward odontoblast differentiation (Choi et al., 2025; Lin et al., 2021; Vogel et al., 2016; Adasooriya et al., 2024; Zhao et al., 2024). This differentiation process can be divided into several key stages (Table 3):

**TABLE 3:** Differentiation process of odontoblasts.

Stage	Key markers	Functional transitions	Refs
Mesenchymal cells near the cervical loop	*Notum, Sall1*	Establish odontogenic competence; Regulate Wnt/BMP signaling; Maintain proliferative potential; Prime commitment	Wen et al. (2020), Lin et al. (2021), Zhao et al. (2024)
Pre-odontoblasts	*Dkk1, Notum*	Fine-tune Wnt signaling (commitment); Initiate extracellular matrix program; Shift toward odontoblast fate	Wen et al. (2020), Zhao et al. (2024)
Early odontoblasts	*Col24a2, Wisp1*	Synthesize dentin matrix components; Organize ECM/maintain limited plasticity; Initiate dentinogenesis	Gong et al. (2022)
Late odontoblasts	*Dmp1, Nupr1, Dspp*	Terminal differentiation; DSPP-dependent mineralization; Polarized dentin matrix secretion	Rangiani et al. (2012), Lavický et al. (2025), Oh et al. (2015)

Mesenchymal Cells near the Cervical Loop: At this stage, mesenchymal cells retain their pluripotent potential. The expression of markers such as *Notum* and *Sall1* not only marks the initial stages of odontoblast differentiation but also regulates the activation of key signaling pathways like Wnt and BMP, which are critical for the subsequent commitment to odontoblast differentiation (Wen et al., 2020; Lin et al., 2021).

Pre-odontoblasts: Pre-odontoblasts show expression of *Dkk1* and *Notum*, signaling a shift toward odontoblast commitment. These markers are involved in regulating the Wnt and BMP pathways, which are pivotal for the maturation of odontoblasts. In this phase, the cells begin to adopt characteristics that will eventually contribute to dentinogenesis, such as initiating extracellular matrix production (Wen et al., 2020; Wu et al., 2017).

Early Odontoblasts: Marked by *Col24a2* and *Wisp1*, early odontoblasts synthesize extracellular matrix components for dentin formation, while retaining multipotency, suggesting they could contribute to other tissue types under certain conditions (Gong et al., 2022).

Late Odontoblasts: Fully differentiated odontoblasts express *Dmp1*, *Nupr1*, and *Dspp*, with DSPP being crucial for dentin mineralization. These cells are functionally specialized in dentin formation and exhibit terminal differentiation (Rangiani et al., 2012; Lavický et al., 2025; Oh et al., 2015).

In human teeth, odontoblasts in immature pulp express genes like *COL3A1, VCAN*, and *FBN2*, essential for extracellular matrix formation during early dentin mineralization (Quispe-Salcedo and Ohshima, 2021). Interestingly, PTN (Pleiotrophin), expressed throughout tooth development, is found at higher levels in teeth with incomplete roots, suggesting its role in root formation and pulp-dentin complex repair (Quispe-Salcedo and Ohshima, 2021; Balzano et al., 2021). As odontoblasts mature, proteins like NES, CLU, and S100A6 are upregulated, contributing to cellular stability and preventing pathological mineralization (Katsura et al., 2014).

Recent scRNA-seq data provide deeper insights into *MAP1B*, a neural marker, and its role in odontoblasts. *MAP1B*, critical in neuronal cytoskeleton formation, regulates spatial organization and development. In odontoblasts, *MAP1B* is expressed beneath the cell body and along processes, suggesting a dual role in odontogenesis and dentin repair after injury (Garner et al., 1990; Tortosa et al., 2011; Cui et al., 2014). Additionally, *SLC12A2, ST8SIA1*, and *TRPM7* play key roles in odontoblast differentiation, ion transport, and osteogenic differentiation, influencing the regenerative capacity of dental pulp stem cells (DPSCs) (Wisit et al., 2006; Nakano et al., 2016; Lee et al., 2019). These findings highlight the multifaceted process of odontoblast differentiation, involving both structural and functional changes.

### Fibroblasts: key players in dental pulp development and repair

Dental fibroblasts (DFs) are the most abundant cell type in the dental pulp and are distributed throughout the pulp tissue, particularly in the multicellular layer (Ducy et al., 1997; Liu and Lee, 2013; Chen et al., 1996); sometimes, they are also called pulp cells. These cells have elongated, spindle-shaped cells with multiple short protruding stellate, darkly stained nuclei, lightly stained and homogeneous cytoplasm, and varied cytosol size depending on their functional state. Microscopically, DFs exhibit abundant rough endoplasmic reticulum and mitochondria, along with well-developed Golgi complexes, indicating active collagen synthesis. They are also capable of degrading the extracellular matrix, which includes type III collagen, proteoglycans, and glycosaminoglycans (Wisit et al., 2006). Fibroblasts, like many other somatic cell types, can be reprogrammed into induced pluripotent stem cells using specific transcription factors, and these iPSCs are capable of differentiating into cells of all three germ layers, including osteoblasts, chondroblasts, adipocytes, muscle and tendon cells, and endothelial cells (Yu et al., 2007; Takahashi et al., 2007).

Given their abundance, dental fibroblasts (DFs) comprise functionally distinct subpopulations with site- and context-specific roles in the pulp microenvironment.

i. Two core DF subsets (Yin et al.) (Yin et al., 2021): one subset is *FOXO3* ^*+*^ */OMD* ^*+*^, coupling oxidative-stress control and cell-cycle/metabolic regulation (*FOXO3*) with matrix mineralization (OMD). The second subset is *SOX4* ^*+*^ */POSTN* ^*+*^, associated with cellular plasticity, migration, and tissue repair. Both subsets participate in cell morphogenesis and differentiation.
ii. Six DF subclusters (Pagella et al.) (Pagella et al., 2021): scRNA-seq annotated six DF subclusters with discrete functions: *CXCL14*-enriched clusters (angiogenic/chemoattractant properties); *PTN* ^*+*^ cluster (mesenchymal signaling linked to odontogenic support); *DLX5*-high cluster (regulation of tooth development); *CTNNB1*-high/*GOLIM4*-high cluster (WNT pathway activity and vesicular trafficking); *OMD* ^*+*^ fibroblasts (matrix mineralization); and *COCH* ^*+*^ fibroblasts (mechanosensing and response to mechanical loading). Notably, *KRT18*, typically epithelial, is also detected in dental pulp fibroblasts, suggesting a tissue-specific role in this niche.

Importantly, even though dental fibroblasts are non-immune cells, they have the highest quantity of ligand-receptor pairs, enabling them actively to participate in pulp inflammation. Jiravejchakul et al. found that *PTN* and *MDK* have tissue-specific high expression in dental pulp (DP) and are key genes that distinguish fibroblasts in DP from other tissues. Furthermore, DFs interact with various dental pulp cells through *PTN* and *MDK*, including MSCs, non-myelinated Schwann cells and other cells, with significant interaction involving Schwann cells and odontoblasts. The receptors for *PTN* and *MDK* produced by fibroblasts vary across different cell types (Jiravejchakul et al., 2023). *IGF2* and collagens are other common ligands expressed by fibroblasts (Yin et al., 2021).

In addition to their varied roles in cellular functions and dental tissue health, DFs can modulate immune responses in the dental pulp. They are the only non-immune cells in dental pulp capable of synthesizing all complement proteins (Chmilewsky et al., 2014). Physical injury/bacterial Stimulating pulp fibroblasts activate the complement system to produce membrane attack complex (MAC)to kill various bacteria, C5a and C3a to recruit pulp progenitor cell recruitment, critical for initiating regenerative processes after dentin-pulp injury, and C3b to inhibit cariogenic bacteria and enhance their phagocytosis (Chmilewsky et al., 2014; Chmilewsky et al., 2013; Jeanneau et al., 2015; Rufas et al., 2016). DFs also communicated with macrophages positively and negatively (Yin et al., 2021) and additionally induce proinflammatory M1-type macrophage differentiation with high bacterial phagocytosis to control infection at the carious front, While DFs located at the periphery of the inflamed area induce anti-inflammatory M2-type macrophage differentiation (Le Fournis et al., 2021). Taken together, DFs are involved in the local control of pulpal inflammation through complement activation and directing the inflammatory response.

### Undifferentiated mesenchymal cells in the dental pulp

Mesenchymal stem cells (MSCs) are a type of multipotent progenitor cells initially isolated from adult bone marrow but later found to be present in other tissues of adults and fetuses. These cells can be isolated from a wide range of tissues, including umbilical cord blood, peripheral blood, adipose tissue, skeletal muscle and amniotic fluid, and dental pulp, where they are also present (Estrela et al., 2011). In dental pulp, when odontoblasts react to mild stimuli by producing reactionary dentin, a more intensive stimulus may cause the death of existing odontoblasts. In such cases, dentin regeneration is mediated by the differentiation of a new generation of odontoblast-like cells from a precursor population, which consists of undifferentiated mesenchymal cells residing in the pulp. Upon external stimulation, these MSCs first migrate to the damaged area, then proliferate and differentiate into dentinogenic cells, which then secrete dentin matrix and eventually form reparative or reactionary dentin (Kitamura, 1999).

Smith has summarized the possible sources of these progenitor cells remaining in pulp: the cell-rich layer of Höhl adjacent to the odontoblasts (Jeanneau et al., 2015), perivascular cells, undifferentiated mesenchymal cells and fibroblasts (Ruch, 1998). Recent studies utilizing single-cell RNA sequencing (scRNA-seq) have confirmed the presence of these MSCs in mature dental pulp tissue in both mice and humans. *THY1/CD90* is one of the most common markers used to define mesenchymal stromal cells (Saalbach and Anderegg, 2019) and is employed to distinguish dental MSCs from dental fibroblasts, although its expression is limited to specific MSC clusters (Pagella et al., 2021). Pagella et al. employed markers such as *FRZB,* being used to label dental follicle cells destined to form the future periodontal tissues in the early developmental stage, and *NOTCH3*, expressed in perivascular mesenchymal stem cells, to effectively identify MSCs in dental pulp (Pagella et al., 2021; Mitsiadis et al., 2017; Wang et al., 2014; Lovschall et al., 2007; Jamal et al., 2015). Other common markers shared by dental MSCs include *ACTA2, TAGLN* and *MYH11* (Pagella et al., 2021; Balic et al., 2023), which are found in smooth muscle cells (Hermans et al., 2022), reflecting the MSCs’ location near blood vessels and their role in forming perivascular networks. MYH11, in particular, has been reported to influence the fate determination of MSCs (Ledesma-Martínez et al., 2016; Dominici et al., 2006; Sharpe, 2016; Talele et al., 2015).

Gene Ontology (GO) enrichment analysis of gene expression patterns during the transition of MSCs in human pulp has revealed that these cells can differentiate into two primary lineages: mineralization, leading to odontoblast formation, and myogenesis, resulting in the formation of pericytes that reside in mature pulp and contribute to tissue repair and defence (Ren et al., 2022; Wong et al., 2015; Armulik et al., 2005). MSCs are intrinsically heterogeneous cell populations, and this heterogeneity is a general feature of MSCs derived from multiple tissues. Recent advances in single-cell RNA sequencing (scRNA-seq) have enabled a more detailed characterization of this inherent heterogeneity, revealing distinct subpopulations of MSCs within the dental pulp, each with unique gene expression profiles and functional properties. Yin et al. found that the dental pulp contained three groups of MSCs by scRNA-seq, and their marker genes were respectively *MYH11, KLF2, TAGLN*; *AKAP12, TMBIM6, SELENOP; CPE, GPX3, NDUFA4L2* (Yin et al., 2021). In another study by Ren et al., data from two adult pulps (Pagella et al., 2021), one young apical papilla pulp (Krivanek et al., 2020), and one young pulp from a 13-year-old boy’s third molar were integrated. MSCs were divided into five subgroups: C1 and C2 primarily express genes related to ionic regulation and cytoskeleton formation; C3 is characterized by the high expression of genes associated with *THY1/CD90,* functions of lipid synthesis, and transport, with higher stemness as well; C4 has an expression profile similar to that of dentin, with both C3 and C4 enriched in BMP receptor binding, indicating their roles in dentin differentiation and mineralization; and C5 is enriched in genes associated with the SAGA complex, dynamin complex, and polysaccharide binding (Ren et al., 2022). Their study also indicated that MSCs in immature pre-eruptive teeth from younger individuals have a higher proportion of C3 cells, which decreases as the tooth matures, suggesting that younger pulp may possess greater differentiation potential.

Although MSCs are present in both the periodontium and dental pulp tissue and share significant homogeneity, MSCs in periodontal tissue tend to adopt a fibroblast-like fate and exhibit long-term high migratory behavior (Schiraldi et al., 2012; Shellard and Mayor, 2019). The different microenvironments cause these differences, which are in (Pagella et al., 2021; Diekwisch, 2002; Luan et al., 2009). In a study comparing DPSCs and PDLCs without *in vitro* culture, MSCs from dental pulp were found to have a higher proportion of cells at the S and G2/M phases (36.1%), indicating a higher proliferation rate (Yang et al., 2024). The diversity and complementary unity of undifferentiated mesenchymal cells in the dental pulp are critical for the repair and maintenance of local homeostasis within the pulp tissue. This diverse cell population structure may also play a key role in achieving functional pulp regeneration.

## Comparision between MSCs in the dental pulp and monolayer cultured human dental pulp stem cells

### Dental pulp stem cells: novel therapeutic stem cells with high heterogeneity

Since the discovery of MSCs, stem cell biology has become an important research direction in tissue engineering and regenerative medicine (Huang et al., 2009). While research on bone marrow-derived MSCs (BMSCs) has been the most extensive, the collection process for BMSCs is painful and yields low cell numbers, prompting researchers to explore alternative sources (Alvarez et al., 2015). A unique population of postnatal dental pulp stem cells (DPSCs) isolated from dental pulp tissue, first identified in 2001, have shown significant potential due to their ability to differentiate into multiple cell types under appropriate conditions (Gronthos et al., 2000). The discovery of DPSCs has greatly expanded the understanding of undifferentiated mesenchymal stem cells within dental pulp. With their angiogenic, neurogenic, and dentinogenic capabilities, DPSCs are promising candidates not only for regenerating the pulp-dentin complex and treating oral diseases (Chen et al., 2016; Xuan et al., 2018; Tanikawa et al., 2020), but also for broader applications in treating systemic diseases (Suda et al., 2022; Wenceslau et al., 2022). Due to their regenerative differentiation potential and ease of acquisition and expansion *in vitro*, DPSCs offer an ideal alternative to BMSCs for therapeutic applications.

DPSC can express a variety of conventional MSCs markers such as *CD13, CD29, CD44, CD73, CD90, CD105, CD106, CD146, CD166, CD271*, while not expressing *CD3, CD8, CD11b (or CD14), CD15, CD19 (or CD79α), CD33, CD34, CD45* (Jo et al., 2007; Pivoriuūnas et al., 2010; Cui et al., 2021). The isolation of specific multipotent DPSC subsets that can lead to the formation of appropriate cell types by identifying specific markers is important for future craniofacial defect repair. These subsets are highly heterogeneous, determined by various surface markers, and exhibit distinct regenerative commitments (Alvarez et al., 2015; Pisciotta et al., 2015). Alvarez et al. (Alvarez et al., 2015) explored the functional significance of various surface marker combinations in isolating homogeneous populations of mesenchymal stem cells (MSCs) from cultured DPSCs. They found that *CD271*-isolated DPSCs exhibited the highest quantity (10.6%) and the strongest odontogenic and chondrogenic potential, suggesting that *CD271* is a critical marker for selecting DPSCs with enhanced capacity to differentiate into dentin- and cartilage-forming cells. This is significant because odontogenesis and chondrogenesis are essential for tooth regeneration and cartilage repair. In contrast, *STR O -1/CD146*-isolated DPSCs, which had the lowest quantity (0.3%), showed a reduced differentiation potential, highlighting that *STR O -1* alone may not be sufficient for isolating high-quality DPSCs with strong differentiation capabilities. Interestingly, DPSCs isolated with *CD51/CD140α* showed the highest quantity (27.3%), as well as a stronger odontogenic and chondrogenic potential compared to other subsets, suggesting that these markers, specifically *CD51* and *CD140α*, may be particularly useful for enhancing the regenerative capacity of DPSCs for both dentin and cartilage tissue engineering. These findings provide valuable insights into how selecting specific markers can optimize the use of DPSCs in clinical regenerative applications, especially in tooth repair and regeneration. Moreover, *ALDH1*, an intracellular enzyme, is potentially associated with cellular stemness, as the stem cell population with high expression of this enzyme exhibits a more pronounced undifferentiated profile. This is based on the isolation of hematopoietic stem cells with high expression of primitive cell and clonogenic progenitor markers through the activity-based separation of *ALDH1* expression (Hess et al., 2006; Moreb, 2008). Machado et al. demonstrated that ALDH1 can be used as a marker for DPSCs, with the expression activity of *ALDH1* in DPSCs is 16.2% (Machado et al., 2016).

scRNA-seq provides new insight into the classification of cultured DPSCs. According to Lee’s work, the DPSCs themselves are a collection of cell populations with different cell types, which contain 0.1% neurons, 15.2% fibroblasts and 58.4% MSCs (Lee et al., 2022). They found that primary cultured DPSCs could be divided into neurogenic, expressing *COL4A1, NEFL, NEFM*, S100A4 and neurofilament assembly bundle-associated gene sets, and osteogenic, expressing *VCAN, FN1, COL1A2, COL1A1* and *DCN* (Lee et al., 2022). Compared to DPSCs, PDLSCs demonstrate better osteogenic potential. Zhang et al. classified hDPSCs into four types by markers, including classical MSCs (70%), fibroblast-like (28%), monocyte-like (2%) and perivascular-like DPSCs (1%). While coculturing the hDPSCs with *Enterococcus faecalis*, the percentage of fibroblast-like DPSCs increases, showing fibroblast differentiation (Zhang W. et al., 2023). *In vitro* cultures of DPSCs are easy to implement and perform well, which is important for clinical applications because regenerative therapies usually require large numbers of stem cells.

### Differences between dental pulp stem cells from deciduous and permanent teeth

Stem cells isolated from human exfoliated deciduous teeth (SHED) and those obtained from permanent teeth (DPSCs) both originate from neural crest cells, yet they exhibit notable differences in their biological characteristics. SHED, derived from younger dental pulp with a less mineralized environment, generally show higher proliferation rates, enhanced neurogenic and angiogenic potential, and lower immunogenicity than DPSCs. These features likely reflect their more immature developmental status and greater cellular plasticity (Miura et al., 2003; Shi et al., 2020).

In contrast, DPSCs from permanent teeth display stronger odontogenic and osteogenic differentiation potential, consistent with their physiological role in maintaining and repairing mineralized dental tissues. These lineage preferences appear to be influenced by the tissue-specific microenvironment and the donor’s age (Lee et al., 2011; Sabbagh et al., 2020). While SHED excel in rapid expansion and neural differentiation, their odontoblastic potential may be relatively limited, suggesting that SHED and DPSCs could be better suited for distinct regenerative purposes.

From a translational perspective, these differences have practical implications. The high proliferative and neurogenic abilities of SHED make them attractive candidates for applications in neural repair, immunomodulation, or vascular regeneration. Conversely, DPSCs, with their robust dentinogenic and osteogenic capacities, may be more appropriate for dentin–pulp complex regeneration or other hard-tissue repair strategies. Recognizing these distinctions is essential for selecting the optimal stem cell source for specific therapeutic contexts and for accurately interpreting outcomes in preclinical studies (Miura et al., 2003; Gronthos et al., 2000; Shi et al., 2020; Lee et al., 2011; Sabbagh et al., 2020).

### *In vivo* or *in vitro*: what changes?

The terms “dental pulp stem cells,” “undifferentiated mesenchymal cells,” and “mesenchymal stromal cells” are often used interchangeably in studies related to dental mesenchymal cells (Song et al., 2023; Li et al., 2023; Ivanovski et al., 2024; Stefańska et al., 2024). However, important distinctions exist between freshly isolated MSCs and cultured cells—the latter being the “true” DPSCs used in most *in vitro* studies. Comparative analysis using scRNA-seq by Cui et al. revealed that *in vitro* culture induces substantial compositional and transcriptional changes compared with freshly isolated DPSCs, which actually comprise a mixture of MSCs and fibroblasts. Among these, *MCAM* ^*+*^ *JAG* ^*+*^ *PDGFRA* ^*-*^ subpopulations retain the greatest transcriptional similarity to their freshly isolated counterparts (Cui et al., 2021). Extended culture enriches *PDGFRA* ^*+*^ cells and promotes osteogenic differentiation, but also introduces senescence-related changes. *In vivo* studies have identified *NG2, Gli1*, and *Celsr1* as key DPSC markers, rarely expressed in conventional *in vitro*–expanded MSCs (Feng et al., 2011; Zhao et al., 2014; Nobre et al., 2021; Parthiban et al., 2020) highlighting potential limitations of extrapolating *in vitro* findings to *in vivo* biology.

Several surface markers are commonly used to characterize DPSCs and their subpopulations, among which *STR O -1, c-Kit,* and *CD34* are the most studied*. STR O -1* marks early mesenchymal precursors with osteogenic and chondrogenic potential (Ferrari et al., 1998; Bianco and Gehron Robey, 2000; Dennis et al., 2002; Gronthos et al., 2003; Shi and Gronthos, 2003; Zhan et al., 2004; Yu et al., 2010; Zhou et al., 2025; Perczel-Kovách et al., 2021), while *c-Kit* is expressed in a range of stem cells, including neural crest–derived lineages (Barclay et al., 1988; Kim et al., 1998; Wehrle-Haller and Weston, 1999; García-Pacheco et al., 2001). *CD34*, classically a hematopoietic marker, can also appear in mesenchymal subsets; *CD34* ^*+*^ DPSCs reportedly show higher proliferation and neurogenic potential than *CD34* ^*−*^ cells; however, *CD34* expression is unstable *in vitro* and diminishes with successive passages. This instability raises a critical concern: using *CD34* as a selection marker for clinical applications may lead to inconsistent cell populations, potentially affecting therapeutic outcomes (Pisciotta et al., 2015; Laino et al., 2006; Lin et al., 2012; Stolzing et al., 2012). Beyond surface markers, *in vitro* culture reshapes the global transcriptional landscape of DPSCs, downregulating genes involved in cell cycle, DNA replication, and repair, and reducing multipotency toward osteogenic, chondrogenic, and adipogenic lineages (Banfi et al., 2002; Flanagan et al., 2017). Expression of other markers such as *CD44, CD271, CD146,* and *CD106* also shifts, with *CD146* upregulated and *CD106* downregulated. These changes collectively alter cellular signaling, migration, and differentiation potential, which may impair the regenerative capacity of DPSCs in clinical contexts. Monoculture conditions, particularly at high passage numbers, can thus compromise the translational relevance of *in vitro*-expanded cells. These differences are summarized in Table 4. Freshly isolated DPSCs better reflect the *in vivo* stem cell niche, while *in vitro* culture induces compositional shifts, senescence, and reduced multipotency. These differences should be carefully considered when interpreting *in vitro* studies and designing regenerative applications.

**TABLE 4:** Comparisons of freshly isolated DPSCs and *in vitro* cultured DPSCs.

Feature/Marker	Freshly isolated DPSCs	In *v*itro cultured DPSCs
Cell composition	Mixture of MSCs and dental fibroblasts	*MCAM* ^*+*^ *JAG* ^*+*^ *PDGFRA* ^*-*^ subpopulations retained; *PDGFRA* ^*+*^ cells enriched
Markers	*NG2* ^*+*^ *, Gli1* ^*+*^ *, Celsr1* ^*+*^ *; STR O -1* ^*+*^ *, c-Kit* ^*+*^ *, CD34* ^*+/−*^	*NG2* ^*-*^ *, Gli1* ^*-*^ *, Celsr1* ^*-*^ *(rare); STR O -1* ^*+*^ (osteogenic only after extensive passaging); *c-Kit* ^*+*^ *; CD34* gradually lost
Cell morphology	Small, spindle-shaped	Larger, flattened
Proliferation capacity	High	Slows with passage; early senescence evident
Multipotency	Osteogenic, chondrogenic, adipogenic, neurogenic	Osteogenic enhanced; overall multipotency reduced
Gene expression	Active cell cycle, DNA replication and repair genes	Downregulation of cell cycle, DNA repair, and replication genes
Migration and signaling	Normal interaction with *in vivo* microenvironment	Impaired; altered signaling and migratory capacity
Senescence and aging	Minimal	Replicative senescence increases with passage
Functional relevance	Reflects *in vivo* DPSC niche	May deviate from *in vivo* phenotype; affects translational relevance

Taken together, these observations have direct implications for regenerative medicine. Low-passage, freshly isolated or minimally cultured DPSCs are likely more reliable for tissue repair applications, while high-passage cells may show reduced efficacy due to senescence and altered gene expression. Markers such as *CD34* should be interpreted cautiously, and functional validation of cultured DPSCs is essential before clinical use. Future single-cell and spatial transcriptomic studies comparing freshly isolated versus cultured, and low- versus high-passage DPSCs, will be critical to optimize selection criteria and culture conditions, ultimately improving the safety and efficacy of MSC-based regenerative therapies.

## Conclusion and future perspectives

As the global population ages, the demand for regenerative medicine and treatments for immune-related diseases has grown increasingly urgent. Stem cell-based therapies are expected to play a pivotal role in addressing a wide range of medical conditions, including those related to ageing and chronic diseases (Hoang et al., 2022). In this context, the dental organ, with its rich reservoir of MSCs, provides a valuable model for studying stem cell biology and its applications in regenerative medicine.

The development of dental tissues, from initiation to maturation, exhibits a high degree of temporal heterogeneity, particularly in its mesenchymal components. A key question that has garnered significant attention is understanding which genes drive these temporally specific transformations of mesenchymal stem cells at various developmental stages. Although scholars may differ slightly in the precise timing of these stages, the division of tooth germ development into the bud, cap, and bell stages is widely accepted. As research continues to deepen, the biological events and regulatory mechanisms governing tooth germ development are being gradually unveiled. Traditional methods for studying tooth development, such as immunostaining, RNAscope, and flow cytometry coupled with knockout or conditional knockout mice, have provided foundational insights.

However, with the increasing sophistication and widespread adoption of high-throughput sequencing technologies, particularly single-cell sequencing, researchers are now able to classify distinct cell clusters at each developmental stage and map their trajectories. This developmental process can be viewed holistically as an expansion from a single point to a complex structure, marked by the continuous differentiation of progenitor cells into terminal cells with distinct functions, thereby optimizing various dental functions (Krivanek et al., 2023).Although scRNA-seq provides detailed insights into cellular heterogeneity, it has several limitations. Spatial context is lost during cell dissociation, lowly expressed genes may go undetected, and transcriptional changes can occur due to tissue processing (Adema et al., 2024). Batch-to-batch variability may also influence the data, and scRNA-seq captures only a snapshot in time. Combining scRNA-seq with spatial transcriptomics, longitudinal studies, or imaging approaches can help overcome these limitations and offer a more complete view of cellular behavior (Baran and Doğan, 2023).

The advancement in studying the key biological events during tooth development and dental pulp maturation allows us to identify molecular targets and guide future research directions. Since the groundbreaking discovery of the biological event where odontogenic potential transitions from dental epithelium to mesenchyme during tooth development, there has been ongoing research into the underlying regulatory mechanisms and potential progenitor cell transformations involved in this process. In recent years, researchers have identified significant changes in gene expression levels before and after the transition of odontogenic potential through high-throughput sequencing technologies (Wang et al., 2022). However, there remains a lack of in-depth studies on the specific functions and regulatory mechanisms represented by these differential genes during this transition. After the transfer of odontogenic potential from epithelium to mesenchyme, the ultimate formation of molars or incisors is determined by the mesenchyme (Kollar and Baird, 1970a). Researchers have found that the earliest differentiation between molars and incisors can be observed in the mesenchyme at E12.5 (Wang et al., 2022). Understanding this process is pivotal in elucidating how epithelial and mesenchymal interactions coordinate during early embryonic development. Furthermore, numerous distinct pathways and spatiotemporally heterogeneous key genes are involved in the development of molars and incisors, and the loss of function in these genes can lead to conditions such as tooth agenesis (Wong et al., 2018) and root development malfunctions (Wang et al., 2023). As teeth develop towards maturity, dental mesenchymal cells with multi-directional differentiation potential have increasingly attracted attention. Due to their ease of *in vitro* acquisition and amplification, as well as their potential to differentiate into adipocytes, chondrocytes, osteoblasts, and neural progenitor cells, researchers hold high hopes for their roles in dental pulp regeneration and even tooth regeneration (Ren et al., 2022; Wong et al., 2015). However, *in vitro* studies have shown that the differentiation potential of these cells diminishes with increasing culture time and passage number, accompanied by signs of cellular ageing (Pisciotta et al., 2015). This undoubtedly impedes further research by MSC in the fields of tissue repair and regenerative medicine. These findings have, in turn, fueled interest in the study of cellular ageing and its implications for regenerative therapies. Despite these challenges, the rapid advancements in technology and conceptual frameworks continue to drive progress in developmental biology and regenerative medicine.

From the initial explorations of morphology and mechanisms to the current studies on subpopulation temporal heterogeneity, biomechanics, *in vitro* transplantation, and more, revolutionary breakthroughs and advancements have been made (Cai et al., 2007; Svandova et al., 2020; Mammoto et al., 2015; Calamari et al., 2018). These established insights can be applied to clinical translation, potentially cracking the genetic developmental code and achieving pulp regeneration and even whole tooth regeneration. In recent years, the application of translational medicine in tooth development research has achieved groundbreaking progress, demonstrating significant clinical potential. The rapid advancement of CRISPR-Cas9 gene editing technology has made it possible to correct gene mutations associated with congenital tooth development abnormalities (Tchasovnikarova et al., 2017). In experimental models, this technology has successfully rectified various genetic defects related to tooth development and holds promise for future application in the treatment of hereditary dental diseases in humans. Concurrently, stem cell technology has made substantial strides in the field of dental tissue regeneration. Pluripotent stem cells (iPSCs) have the ability to differentiate into various cell types. Scholars have successfully induced dental-derived mesenchymal cells into iPSCs (Zhu et al., 2019; Tamaoki et al., 2010; Yan et al., 2010). By applying the insights gained from studies on the spatiotemporal specificity of dental mesenchyme, we can utilize these key regulatory genes to induce iPSCs. This approach might successfully guide them to differentiate into dentin, enamel, and periodontal tissues, ultimately creating functional tooth structures *in vivo* and realizing the goal of tooth regeneration (Gao et al., 2022). This advancement offers a viable solution for the treatment of complex tooth defects and dental pulp injuries.

Furthermore, dental developmental abnormalities often manifest as part of broader systemic diseases. The integration of single-cell sequencing technology with gene detection methods offers a powerful tool to pinpoint specific cell populations, developmental stages, and genetic alterations associated with these abnormalities. Advancements in single-cell sequencing have enabled precise analysis of the tooth development process, allowing researchers to map gene expression profiles across different cell types during various stages of tooth formation. This comprehensive mapping has led to the identification of critical genes and signaling pathways, laying the groundwork for personalized treatment strategies. By combining these insights with CRISPR-Cas9 gene editing technology and tissue engineering techniques, future research is poised to design more targeted therapeutic approaches (Datlinger et al., 2017; Kim et al., 2024). These approaches will be tailored to individual patients’ genetic backgrounds and specific tooth development characteristics, significantly improving treatment efficacy and reducing adverse effects.

Although the majority of transcriptomic insights into tooth development have been obtained from mouse models, recent studies have applied single-cell RNA sequencing to early human tooth germs. These works have delineated cellular heterogeneity and developmental trajectories in human teeth, offering complementary insights into species-specific regulatory programs and differentiation pathways (Zhang et al., 2025; Yin et al., 2021; Pagella et al., 2021; Jiravejchakul et al., 2023; Ren et al., 2022; Cui et al., 2021; Lee et al., 2022; Shi et al., 2021; Weng et al., 2025; Gu et al., 2025; Shan et al., 2025; Qian et al., 2023). Considering the scope and focus of the present review, these human studies are not discussed in detail; however, we include them as references to encourage readers to consult these resources for a more comprehensive understanding of human tooth development.

In conclusion, while substantial progress has been made in understanding the development and potential of dental mesenchymal stem cells, much work remains ahead. The inherent diversity and complementary functions of these undifferentiated cells within the dental pulp could be crucial in unlocking the full potential of regenerative medicine. Continued research in this area is essential to harness their capabilities for clinical applications fully.
